# Who Self-Weighs and What Do They Gain From It? A Retrospective Comparison Between Smart Scale Users and the General Population in England

**DOI:** 10.2196/jmir.4767

**Published:** 2016-01-21

**Authors:** Matthew Sperrin, Helen Rushton, William G Dixon, Alexis Normand, Joffrey Villard, Angela Chieh, Iain Buchan

**Affiliations:** ^1^ Health e-Research Centre, Farr Institute Institute of Population Health, Manchester Academic Health Science Centre University of Manchester Manchester United Kingdom; ^2^ Arthritis Research UK Centre for Epidemiology, Centre for Musculoskeletal Research Institute of Inflammation and Repair, Manchester Academic Health Science Centre University of Manchester Manchester United Kingdom; ^3^ Rheumatology Department Salford Royal NHS Foundation Trust Salford United Kingdom; ^4^ Withings Issy-les-Moulineaux France

**Keywords:** weight gain, weight loss, body weight, body mass index, self-monitoring, connected health technologies

## Abstract

**Background:**

Digital self-monitoring, particularly of weight, is increasingly prevalent. The associated data could be reused for clinical and research purposes.

**Objective:**

The aim was to compare participants who use connected smart scale technologies with the general population and explore how use of smart scale technology affects, or is affected by, weight change.

**Methods:**

This was a retrospective study comparing 2 databases: (1) the longitudinal height and weight measurement database of smart scale users and (2) the Health Survey for England, a cross-sectional survey of the general population in England. Baseline comparison was of body mass index (BMI) in the 2 databases via a regression model. For exploring engagement with the technology, two analyses were performed: (1) a regression model of BMI change predicted by measures of engagement and (2) a recurrent event survival analysis with instantaneous probability of a subsequent self-weighing predicted by previous BMI change.

**Results:**

Among women, users of self-weighing technology had a mean BMI of 1.62 kg/m^2^ (95% CI 1.03-2.22) lower than the general population (of the same age and height) (*P*<.001). Among men, users had a mean BMI of 1.26 kg/m^2^ (95% CI 0.84-1.69) greater than the general population (of the same age and height) (*P*<.001). Reduction in BMI was independently associated with greater engagement with self-weighing. Self-weighing events were more likely when users had recently reduced their BMI.

**Conclusions:**

Users of self-weighing technology are a selected sample of the general population and this must be accounted for in studies that employ these data. Engagement with self-weighing is associated with recent weight change; more research is needed to understand the extent to which weight change encourages closer monitoring versus closer monitoring driving the weight change. The concept of isolated measures needs to give way to one of connected health metrics.

## Introduction

Self-monitoring of weight has a long history rooted in consumer demand for weight control, reinforced in recent decades by public concern over rising obesity levels [1,2]. Frequent self-weighing is associated with weight loss [3,4] and there is evidence of a dose-response relationship with more frequent weighing associated with higher weight loss [5-7]. Technologies that enable weight to be captured digitally and fed automatically into consumer health records can enhance both the utilization and effects of self-weighing [8,9]. This is an example of *connected health technology*: the application of technology to help individuals and their health care providers monitor and maintain health [10].

Data from connected health technologies have potential for adoption in clinical practice and research; however, there are at least 2 concerns with their use. The first concern is that, on an individual level, the accuracy of the data may be considered inferior to that recorded by a health professional. Generally, self-measured height is overestimated and weight is underestimated [11-13]; however, the extent of this is minor and use of self-reported height and weight is considered valid [14-16]. Recall bias may also apply to historical weight measures. A connected health approach may overcome this concern as automatic transfer of data from the weighing device to the consumer health record bypasses reporting and recall bias. The second concern applies on an epidemiological level: there is inherent selection bias in the individuals who choose to self-monitor, so it is difficult to draw population-wide conclusions. Existing literature focuses on participants who volunteered for self-weighing; therefore, the organic uptake of self-weighing remains relatively unexplored.

The aim of this study was to explore the possibility of using data collected from contemporary self-weighing smart scales for epidemiological research. Our first objective was to compare the population of people using smart scales in England with the wider population to get an idea of the selection bias. Our second objective was to understand how engagement with the smart scales varies between participants and how this engagement affects (or is affected by) weight change.

## Methods

### Data

There were 2 sources of data used in this study. The first dataset was the 2011 wave of the Health Survey for England (HSE), used to obtain a representation of the distribution of height, weight, and body mass index (BMI) in England. The HSE is a series of annual cross-sectional surveys carried out in England. First piloted in 1991, it has been fully running since 1992. Weight is measured by a nurse to the nearest 100 g using an electronic scale after removal of shoes or bulky clothing (participants were not weighed if they were pregnant, unsteady on their feet, or chair-bound). Height, to the nearest millimeter, is measured by a nurse using a portable stadiometer. Previous surveys reported, on average, 70% of households agreed to an interview and BMI was available from approximately 90% of those interviewed (with some variation by year and region) [17].

**Figure 1:**
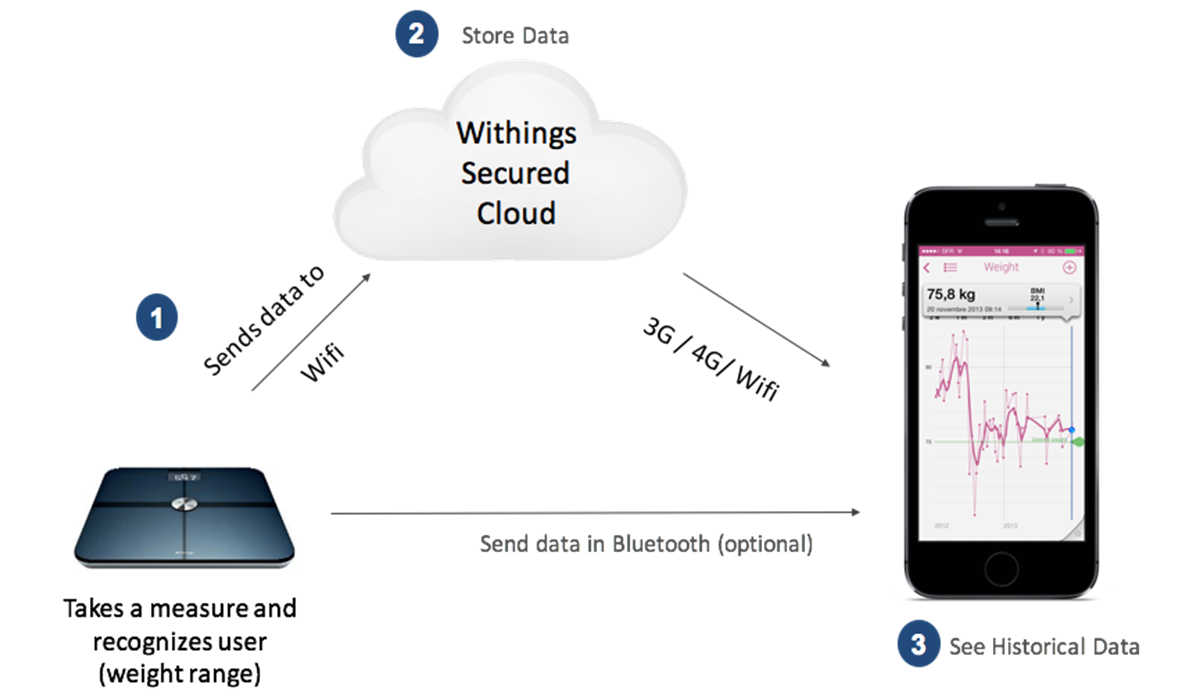
Description of the self-weighing process and data storage for Withings Smart Scale.

The second data source was a random sample of Withings Smart Scale users based in England, representing the population engaged with self-weighing. A *user* is defined as someone who obtained a Withings Smart Scale and created an account under which their measurements are stored. Scales were self-purchased by potential users from retail stores or from the Withings website. The process of a self-weighing and the data being stored is described in Figure 1. A random sample was generated from all users with at least one self-weighing; the full dataset of English Withings Smart Scale users could not be used due to commercial sensitivity; however, the random sample was large enough to afford reasonable contrasts in demographic characteristics and BMI. The follow-up time for a Withings Smart Scale user was defined as the time interval between the first and last available measurement.

The anonymized HSE is publicly available for research purposes. The Withings Smart Scale users consented to their data being used for research purposes as part of the Terms and Conditions when setting up a user account (see [18]).

We restricted analysis a priori to persons aged 16 or older. BMI measurements below 15 and above 70 were assumed to be erroneous and were removed. BMI was used as a continuous variable as well as a categorical variable using the World Health Organization cut-offs [19].

### Statistical Methods

Descriptive statistics were produced using standard methods. We compared these between the 2 datasets (HSE and Withings Smart Scale) and additionally stratified this comparison by gender. Continuous variables that were not expected a priori to have substantial skew (age, height, weight, and BMI) were summarized using means and standard deviations, and compared using *t* tests. Continuous variables that were expected to be skewed (measurements per person, follow-up days, and measurements per person per month) were summarized using medians and the interquartile range. Categorical variables were summarized by the counts and proportions of participants in each group and compared using Fisher exact tests.

We compared the BMI of the smart scale users with the HSE participants using linear regression, with BMI as the response and an indicator of smart scale user as the predictor of primary interest.

Withings Smart Scale data were investigated in more detail to explore the association between engagement with self-weighing and BMI. First, determinants of BMI change over the follow-up period were examined using linear regression. BMI change (the response) was calculated as a single measurement for each individual as the difference between the first and last BMI measures reported divided by the time (in months) between them, with negative change representing overall BMI loss. Individuals required at least 2 measurements to be included in this model. Primary predictors of interest were number of measurements per month, total follow-up time, and initial weight. Second, a multilevel Cox proportional hazard model was used to assess determinants of a weighing event occurring. This was treated as a recurrent event with frailty terms used to account for within-person correlation. The primary covariates of interest for this model were BMI at the previous reading and a measure of the recent change in BMI. Recent change in BMI was considered in 2 ways in 2 separate analyses. The “current” incremental change was defined as the difference in BMI between the previous weighing and the current weighing. This may represent an individual’s perception of recent weight change when making the current weighing. The “previous” incremental change was defined as the difference in BMI between the 2 previous weighings. Therefore, this represents a BMI change that has already been observed before the current weighing. For both measures of change, we recorded whether this was a gain or loss and this was represented in 2 separate variables. For example, if BMI at weighing *t* minus BMI at time *t–1* equalled –0.3, this was recorded as a BMI loss of 0.3 (and the variable for BMI gain was set to zero). We also included an indicator variable of whether BMI was lost or gained. This allowed for some flexibility in modeling the BMI change: a discontinuity at a BMI change of zero represented by the indicator variable and different slopes depending on whether BMI was gained or lost. For these models, the time interval between the first 2 BMI measurements was excluded because the previous change variable was not available; all other time intervals were included. Consequently, individuals required at least 3 measurements to contribute to this model.

For all the preceding models, height, age, and age squared (age^2^) were included as confounders because they are all known to be associated with BMI [1,20]. Separate models were fitted for men and women because it was known a priori that BMI should be interpreted differently for each gender [20]. All analyses were carried out using Stata version 13 software.

## Results

**Figure 2:**
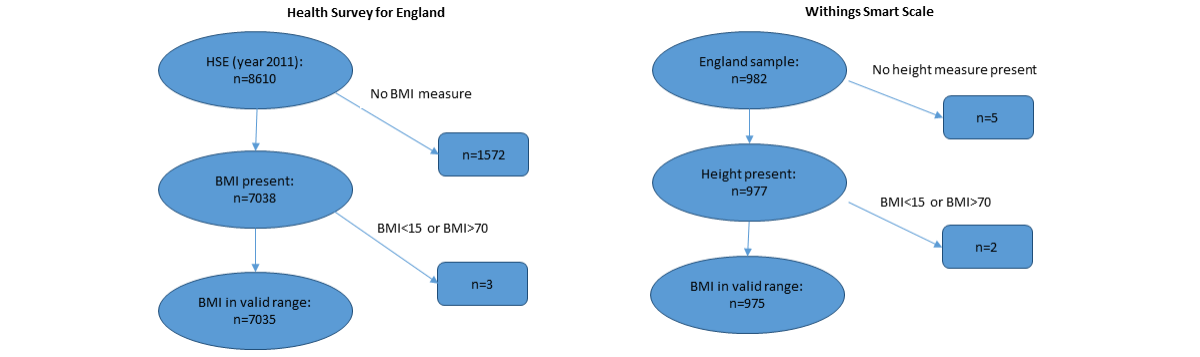
Data exclusion flowchart for Health Survey for England data (left) and Withings Smart Scale data (right).

For the Withings Smart Scale data, there were 975 users in the sample; for the HSE data there were 7035 individuals. A data exclusion flowchart is given in Figure 2.

**Figure 3:**
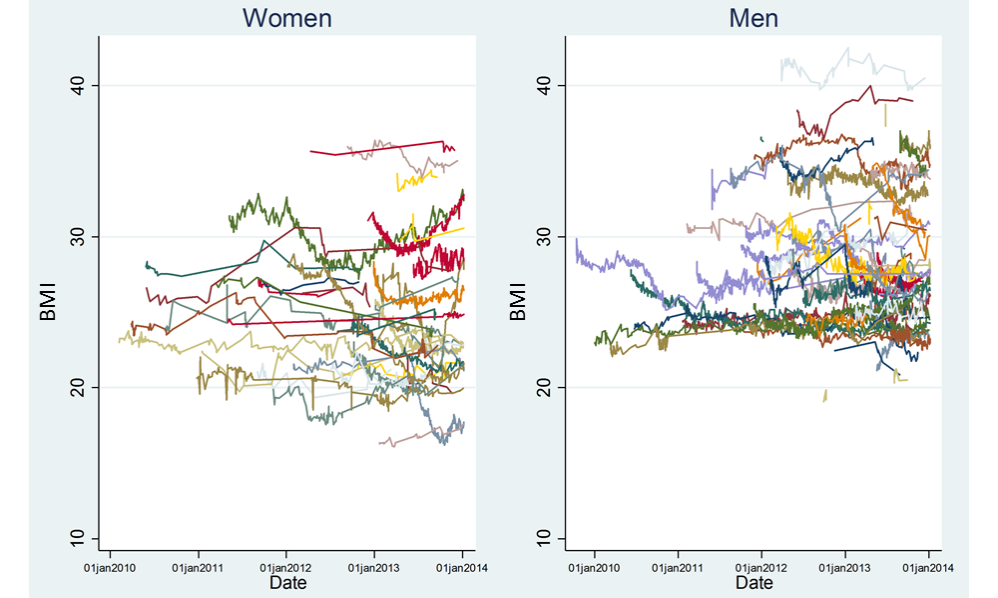
Example BMI trajectories of the first 100 men and 100 women in the Withings Smart Scale data over time (January 1, 2010 to January 1, 2014).

The baseline characteristics of the 2 populations are given in Table 1; this used the first recorded height and weight measurement for each individual in the Withings Smart Scale data. The Withings Smart Scale data contained more men and a younger population than the HSE. For the Withings Smart Scale data, the median follow-up was 377 (IQR 187-700) days for men and 351 (IQR 143-655) days for women, with a median of 87 (IQR 30-188) weighings per man over the entire follow-up period (median 7.6, IQR 3.7-16.1 per month) and median 50 (IQR 15-123) per woman over the entire follow-up period (median 5.5, IQR 2.2-14.1 per month). Example trajectories from the Withings Smart Scale data are also visualized in Figure 3.

**Table 1:** Comparison of baseline characteristics between Withings Smart Scale and Health Survey for England (HSE) participants (N=8010).

Variable		Smart scale n=975			HSE n=7035			*P* ^a^		
		Men	Women	Overall	Men	Women	Overall	Men	Women	Overall
Participants, n (%)		591 (60.6)	384 (39.4)		3164 (44.98)	3871 (55.02)				<.001
Age (years), mean (SD)		39.00 (10.52)	39.34 (12.55)	39.13 (11.36)	49.30 (18.11)	48.86 (18.36)	49.05 (18.25)	<.001	<.001	<.001
Measurements per person, median (IQR)		87 (30-188)	50 (15-123)							
Follow-up days, median (IQR)		377 (187-700)	351 (143-655)							
Measurements per person per month, median (IQR)		7.6 (3.7-16.1)	5.5 (2.2-14.1)							
BMI at first measurement (kg/m^2^), mean (SD)		28.32 (5.42)	25.17 (5.34)	27.08 (5.60)	27.51 (4.79)	27.30 (5.77)	27.39 (5.35)	<.001	<.001	.09
Height (cm), mean (SD)		178.91 (7.77)	165.19 (6.47)	173.51 (9.90)	175.05 (7.42)	161.62 (6.81)	167.66 (9.74)	<.001	<.001	<.001
Weight (kg), mean (SD)		90.65 (18.27)	68.77 (15.62)	82.03 (20.31)	84.37 (15.80)	71.29 (15.58)	77.17 (16.98)	<.001	.003	<.001
**BMI (kg/m** ^**2**^ **), n (%)**										
	Underweight (<18.5)	4 (0.7)	14 (3.6)	18 (1.8)	31 (0.98)	77 (1.99)	108 (1.54)	.01	<.001	.12
	Normal (18.5-24.9)	160 (27.1)	213 (55.5)	373 (38.3)	966 (30.53)	1474 (38.08)	2440 (34.68)			
	Overweight (25.0-29.9)	241 (40.8)	99 (25.8)	340 (34.9)	1373 (43.39)	1286 (33.22)	2659 (37.80)			
	Obese (≥30)	186 (31.5)	58 (15.1)	244 (25.0)	794 (25.09)	1034 (26.71)	1828 (25.98)			

^a^ Based on Fisher exact test or *t* test.

**Figure 4:**
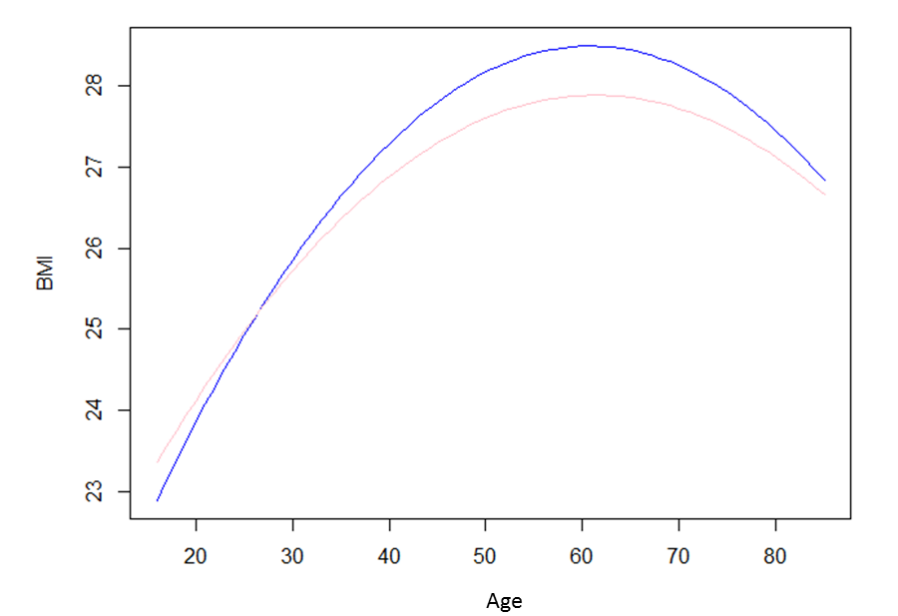
Model-estimated BMI for Health Survey for England data for men of average height (175 cm; blue line) and women of average height (162 cm; pink line).

The regression model for Withings Smart Scale user status on BMI is given in Table 2. Among women, after correction for potential confounders, Withings Smart Scale users had a mean BMI of 1.62 (95% CI 1.03-2.22) lower than the general population (of the same age and height) (*P*<.001). The opposite pattern was seen among male Withings Smart Scale users, who had a mean BMI of 1.26 (95% CI 0.84-1.69) greater than the general population (of the same age and height) (*P*<.001). The results from both samples also corroborated that shorter men and women tend to have higher BMI (reflected in the negative coefficient for height in Table 2). There is a quadratic relationship between BMI and age with BMI generally increasing up to age 60 years then declining (see Figure 4).

**Table 2:** Results of regression model comparing BMI between Withings Smart Scale and Health Survey for England (HSE) data.

Variable	Men, n=3755		Women, n=4255	
	Coef (95% CI)	*P*	Coef (95% CI)	*P*
Smart scale cohort indicator	1.26 (0.84, 1.69)	<.001	–1.62 (–2.22, 1.03)	<.001
Age	0.34 (0.29, 0.39)	<.001	0.27 (0.22, 0.32)	<.001
Age^2^	–0.0028 (–0.0033, –0.0023)	<.001	–0.0022 (–0.0027, –0.0017)	<.001
Height	–0.03 (–0.05, –0.01)	<.001	–0.07 (–0.094, –0.04)	<.001
Intercept	23.42 (19.60, 27.65)	<.001	30.94 (26.55, 35.34)	<.001

We then looked in more detail at the Withings Smart Scale data to understand how engagement with the smart scale technology related to BMI change over time. First, in the regression of BMI change against measurement intensity, we found that more frequent measurement over the entire period was associated with greater weight loss per month in both women (regression coefficient 0.03, 95% CI 0.02-0.05 kg/m^2^ per measurement per month, *P*=.01) and men (regression coefficient 0.03, 95% CI 0.01-0.05 kg/m^2^ per measurement per month, *P*<.001). To put this in context, a man with the median follow-up of 377 days (12.4 months, from Table 1) would be expected to lose 0.37 kg/m^2^ more over the follow-up period than a man with one fewer measurement per month; this is equivalent to 1.13 kg for a man of average height (175 cm). Similarly, a woman with median follow-up of 355 days (11.7 months, from Table 1) would be expected to lose 0.35 kg/m^2^ more over the follow-up than a woman with one fewer measurement per month; this is equivalent to 0.92 kg for a woman of average height (162cm). Higher initial BMI led to a greater reduction per month. See Table 3 for the full regression results.

**Table 3:** Results of regression model for weight loss versus measurement intensity.

Variable	Men, n=586		Women, n=376	
	Coef (95% CI)	*P*	Coef (95% CI)	*P*
Measurements per month	–0.03 (–0.05, –0.02)	<.001	–0.03 (–0.05, –0.01)	.01
Time observed (months)	0.006 (–0.006, 0.018)	.30	0.01 (–0.004, 0.031)	.12
BMI at start	–0.12 (–0.15, –0.09)	<.001	–0.05 (–0.09, –0.02)	.005
Intercept	–5.71 (–9.89, –1.54)	.007	–3.07 (–8.65, 2.50)	.28
Age	0.02 (–0.07, 0.11)	.65	0.08 (–0.004, 0.17)	.06
Age^2^	–0.0002 (–0.0012, 0.0009)	.76	–0.0010 (–0.0020, 0.0000)	.049
Height (m)	2.75 (0.66, 4.85)	.01	0.77 (–2.43, 3.96)	.64

We then considered longitudinal patterns of subsequent weighings based on recent weight change. The results of these analyses are summarized in Table 4. Because at least 3 measurements were required for these models, 9 men and 13 women who had only 1 or 2 measurements each were removed. For the current measure of weight change (see Methods), we found recent weight loss encouraged a subsequent measurement, with hazard ratio (HR) 7.38 (95% CI 7.03-7.75) per unit BMI in men (ie, propensity for weighing is 7.38 times higher for a man whose BMI has dropped 1 kg/m^2^ compared with a man who has remained the same weight) and HR 5.86 (95% CI 5.50-6.25) per unit BMI in women. For the previous measure of weight change (see Methods), weight loss encouraged subsequent measurements but to a lesser extent, with HR 2.88 (95% CI 2.74-3.02) in men and HR 2.44 (95% CI 2.28-2.60) in women. On the other hand, recent weight gain discouraged subsequent measurements in both men and women. Under the current measure of weight gain, HR 0.09 (95% CI 0.09-0.10) was observed for men and HR 0.10 (95% CI 0.09-0.10) was observed for women. For the recent measure of weight gain, smaller effects were observed, but in the same direction with HR 0.41 (95% CI 0.40-0.43) in men and HR 0.40 (95% CI 0.38-0.42) in women. The fact that in all cases the effect was more pronounced for the current incremental change suggests that perceived recent weight change is more important than measured historical weight change as a predictor of further weighing.

**Table 4:** Hazard ratios (HR) calculated from the Cox proportional hazards model.

Variable		Men (88,769 observations on 575 participants)		Women (41,894 observations on 363 participants)	
		HR (95% CI)	*P*	HR (95% CI)	*P*
**Current change**					
	BMI	0.99 (0.98-0.99)	<.001	1.02 (1.01-1.02)	<.001
	Time since first weighing (months)	0.98 (0.97-0.98)	<.001	0.98 (0.98-0.98)	<.001
	Indicates BMI lost	1.20 (1.18-1.22)	<.001	1.06 (1.03-1.09)	<.001
	BMI change (gain)	0.09 (0.09-0.10)	<.001	0.10 (0.09-0.10)	<.001
	BMI change (loss)	7.38 (7.03-7.75)	<.001	5.86 (5.50-6.25)	<.001
	Age	1.03 (1.03-1.04)	<.001	1.01 (1.01-1.02)	<.001
	Age^2^	0.9997 (0.9996-0.9997)	<.001	0.9999 (0.9999-1.0000)	.28
	Height (m)	1.79 (1.63-1.96)	<.001	1.08 (0.93-1.26)	.30
**Previous change**					
	BMI	0.97 (0.97-0.97)	<.001	1.00 (1.00-1.00)	.30
	Time since first weighing (months)	0.97 (0.97-0.97)	<.001	0.97 (0.97-0.97)	.002
	Indicates BMI lost	1.15 (1.12-1.17)	<.001	0.98 (0.95-1.001)	.12
	BMI change where BMI gained	0.41 (0.40-0.43)	<.001	0.40 (0.38-0.42)	<.001
	BMI change where BMI lost	2.88 (2.74-3.02)	<.001	2.44 (2.28-2.60)	<.001
	Age	1.05 (1.04-1.05)	<.001	1.02 (1.02-1.03)	<.001
	Age^2^	0.9996 (0.9995-0.9996)	<.001	0.9999 (0.9998-0.9999)	<.001
	Height (m)	1.47 (1.34-1.61)	<.001	1.18 (1.01-1.37)	.04

## Discussion

### Summary

This study compared English users of Withings smart scales connected to consumer health records to the general population in England. We found that Withings Smart Scale users are younger and more likely to be male than the general population in England. Among women, we found Withings Smart Scale users had, after correction for confounding, a BMI 1.62 kg/m^2^ lower than the general population; for a woman of average height (162 cm), this is a weight difference of 4.25 kg. Among men, we found Withings Smart Scale users had, after correction, a BMI 1.26 kg/m^2^ higher than the general population; for a man of average height (175 cm), this is a weight difference of 3.86 kg. Looking in more detail at Withings Smart Scale users, we found that more frequent measurement was associated with greater weight loss; again considering average height, each additional weighing per month was associated with further weight loss over the entire follow-up period of 1.13 kg for men and 0.92 kg for women. A positive feedback loop was identified in which a recent observed decrease in weight encourages further weighing.

### Strengths and Limitations

A strength of the study is that we used data from large, robust sources for both the general population and the randomly selected population of individuals who use a popular brand of smart scales to monitor their weight. We employed advanced modeling techniques, including multilevel Cox regression, to exploit the longitudinal richness of the data.

A limitation is that the BMI comparison is based on standardized measurement in HSE, whereas readings in the Withings Smart Scale data were not standardized to such things as the amount of clothing worn. However, even self-reported height and weight without automated data capture from one type of instrument are generally accepted to be sufficiently accurate for such comparisons to be made [14]. However, the height data are nonstandardized self-reports recorded into the consumer’s online health record.

The HSE is a cross-sectional study and the Withings Smart Scale data are longitudinal. Therefore, there is a difference in timeframe, although this was minimized by using the 2011 wave of HSE, which is within the Withings Smart Scale data timeframe. Although changes in BMI in the English population are likely to be small over the Withings Smart Scale data timeframe (2010-2013) [1], changes over time in the Withings Smart Scale data could be larger, especially because the use of smart scales has become more widespread over the period. A future study will consider the emergence of use of smart scales over time and test the hypothesis that the smart scale user population converges to the general population over time.

A further limitation is that this is an observational study, so propensity to use self-weighing technology is subject to confounding. We have mitigated this by correcting our comparative models for age, gender, and height. However, we could not consider unmeasured potential confounding factors. An important unmeasured confounder is baseline engagement with weight or BMI; it is likely that individuals with more interest in BMI monitoring are more likely to purchase self-weighing technology, which would amplify the association of smart scale use with BMI control. Therefore, the results of our study should not be interpreted causally and further studies are needed to isolate the causal effect of self-weighing.

### Comparison With Existing Literature

Our findings reinforce those of others that found increased engagement with self-weighing is associated with greater weight loss or reduced weight gain [3-7,21-23]. However, to the best of our knowledge, all existing studies concern participants in weight control programs. Therefore, our study adds to the literature because it demonstrates this effect in a population of smart scale users who may or may not be engaging in weight control programs. In addition, we have uncovered a positive feedback loop in which a weighing showing a decrease in weight encourages a further weighing in the near future.

Unlike other studies, our observations suggest that women engaging with self-weighing technology tend to be lighter than average, whereas men tend to be heavier. A possible hypothesis for this finding could be that men who engage may be fit with high muscle mass.

### Implications for Research/Practice

Users of Withings Smart Scale devices are not representative of the general population. Any inferences about the general population should be corrected for at least age and gender by regression analysis or reweighting. In addition, even after correction for age and gender, BMI measures differ between the smart scales and the general population. Because this difference is in the opposite direction for men and women, there may be complementary reasons for engagement with smart scales between the genders. Further qualitative research into these drivers may allow for transfer across the genders and improve uptake of such devices.

Connected health technologies incorporating self-weighing can provide richer data than those from infrequent contact with health professionals. In particular, much higher longitudinal resolution of BMI can be captured for individuals and populations. However, these data are complex: the relation between the frequency of self-weighing and the underlying level and change in the weight itself needs careful consideration. Usefully, self-weighing is associated with better weight control; however, more research is needed to examine potential mediators and confounders of this relationship.

As personal health records start to gather data from a wider ecosystem of frequent measurement, the links between health observations and behaviors will become more tightly coupled. For example, physical activity monitoring from smart watches linked to weight measures from smart scales brings together information on weight control interventions and outcomes in a potentially persuasive ensemble. The statistical challenges of harnessing linked observation, intervention, and outcome processes should not be underestimated.

Connected health ecosystems are being driven by the consumer health/wellness market, but they also have the potential to support clinical interventions and research [24,25]. At present, such technologies are not ubiquitous; therefore, the selection biases due to the characteristics of those who opt to buy and use them must be considered.

The use of connected health technologies is a promising area for clinical research and practice as well as consumer health markets. Their real potential may be realized through their linkage with each other and with more conventional sources such as electronic health records.

### Conclusion

In this paper, we have demonstrated that current engagement with smart scale technology involves a selected population. Therefore, use of the associated data needs to correct for this selection. We have also demonstrated an opposing selection effect between men and women, with male users being heavier than average and female users being lighter, as well as a positive feedback loop with more frequent weighings following greater weight loss. The drivers behind these findings need to be explored in more detail to understand how engagement with smart scale technology drives, and is driven by, healthy behavior.

## Abbreviations

BMI: body mass index
HR: hazard ratio
HSE: Health Survey for England
